# Efficacy and safety of Xihuang pill for lung cancer: A protocol for systematic review and meta-analysis

**DOI:** 10.1097/MD.0000000000022516

**Published:** 2020-10-09

**Authors:** Junwei Wang, Daorui Hou, Yahui Peng, Jian Xiong, Lu Xiong

**Affiliations:** a Department of Traditional Chinese Medicine Oncology, Guang’anmen Hospital South District; b Department of Traditional Chinese Medicine Oncology, The First People's Hospital of Xiangtan City, Xiangtan, Hunan Province; c Beijing Shahe Hospital; d Department of Oncology, Guang’anmen Hospital, Beijing, China.

**Keywords:** lung cancer, protocol, systematic review and meta-analysis, Xihuang pill

## Abstract

**Background:**

Xihuang pill, a famous traditional Chinese medicine formulation, is a broad-spectrum anti-tumor drug and has been widely used for the treatment of lung cancer in China. The aim of this study is to systematically investigate the efficacy and safety of Xihuang pill for the treatment of lung cancer.

**Methods:**

We will perform the comprehensive literature search in the following databases from their inceptions to August 2020 for data extraction: PubMed, the Cochrane Library, Embase, the China National Knowledge Infrastructure, Wanfang Database, Chinese Science and Technology Periodical Database, and Chinese Biomedical Literature Database. Cochrane Risk of Bias tool will be used to assess the risk of bias of included studies. The RevMan 5.4 and Stata 16.0 software will be applied for statistical analyses. Statistical heterogeneity will be computed by *I*^2^ tests. Sensitivity analysis will be conducted to evaluate the stability of the results. The publication bias will be evaluated by funnel plots and Egger test. The quality of evidence will be assessed by the Grading of Recommendations Assessment, Development and Evaluate system (GRADE) system.

**Results:**

The results of our research will be published in a peer-reviewed journal.

**Conclusion:**

The conclusion of this study will provide evidence to show whether Xihuang pill is an effective intervention for patient with lung cancer.

**OSF registration number:**

10.17605/OSF.IO/W2GHN

## 1 Introduction

Lung cancer is the most commonly diagnosed cancer and the leading cause of cancer death in the world.^[1]^ The annual diagnosis rate of new cases is approximately 1.6 million globally.^[2]^ Treatment options for lung cancer include surgery, radiation therapy, chemotherapy, targeted therapy, and immunotherapy. Despite the improvements in diagnosis and therapy made during the past 25 years, the prognosis for patients with lung cancer is still unsatisfactory.^[3]^

Traditional Chinese medicine is one of the popular alternative treatments for cancer.^[4]^ It is accepted in China to enhance the antitumor effects of conventional therapies, reduce the toxicity of chemotherapy and radiotherapy, alleviate tumor-induced clinical symptoms and cancer pain, and prolong the survival time of postoperational and advanced-stage cancer patients.^[5]^

Xihuang pill, a famous traditional Chinese medicine formulation, has been used to treat tumor-related diseases in China for >100 years.^[6,7]^ Xihuang pill, mainly composed of Ru Xiang (*Olibanun*), Mo Yao (*Myrrha*), She Xiang (*Moschus*), and Niu Huang (*Bovis Calculus*), was approved by the National Medical Products Administration (NMPA) for tumor treatment with approval number Z11020073. Some of the compounds found in these ingredients exert multiple antitumor effects and may synergize with the other ingredients.^[7]^ Xihuang pill can also inhibit the growth of tumor cells and cancer stem cells, prevent tumor invasion and angiogenesis, and regulate the tumor microenvironment. It has exhibited antitumor effects on various cancers such as lung cancer,^[6]^ breast cancer,^[8]^ colorectal cancer,^[9]^ non-Hodgkin lymphoma,^[10]^ and liver cancer.^[11]^

However, there is still a lack of high-quality evidence to support the effectiveness and safety of Xihuang pill on patients with lung cancer. In this work, we will perform a systematic review to evaluate the efficacy and safety of Xihuang pill in the treatment of lung cancer and to provide a reference for clinical application.

## 2 Methods and analysis

This study was prospectively registered in the Open Science Framework (OSF) with a DOI: 10.17605/OSF.IO/4ZUXC. It will be carried out under the guideline of Preferred Reporting Items for Systematic Reviews and Meta-analyses Protocols (PRISMA).^[12]^

### 2.1 Inclusion criteria

#### 2.1.1 Type of study

All published clinical randomized controlled trials (RCTs) of Xihuang pill for the treatment of lung cancer will be included without language restriction. Observational studies, cross-sectional studies, cross-over studies, quasi-RCT, conference abstracts, animal experiments, review articles, and letters will be excluded.

#### 2.1.2 Types of participants

We will include RCTs on participants who are diagnosed as lung cancer. There are no restrictions on research subjects age, sex, race, condition duration, or intensity.

#### 2.1.3 Types of interventions

Studies using Xihuang pill alone or combinations with other interventions to treat the lung cancer will be included. There will be no restriction about the doses and methods of use of intervention. When Xihuang pill used as combinations with other treatments, the control group should also receive the same combination treatments.

#### 2.1.4 Types of outcomes

(1) Primary outcomes:
  Overall survival; progression-free survival.
(2) Secondary outcomes:
  Overall response rate; disease control rate; health-related quality of life; adverse events.

### 2.2 Search strategy

Two researchers will independently search the following databases: PubMed, the Cochrane Library, Embase, the China National Knowledge Infrastructure, Wanfang Database, Chinese Science and Technology Periodical Database, and Chinese Biomedical Literature Database. All the above databases will be searched from the available date of inception until the latest issue (August 2020). No language or publication restriction will be used. Google scholar, Bing scholar, and Baidu scholar will also be retrieved to find out other related literature. In addition, we will search grey literatures to avoid missing any potential studies, such as dissertations, ongoing trials from clinical trials registries, conference abstracts, and reference lists of associated reviews. An example of search strategy for PubMed database that combines MeSH terms and free words will be adopted. The search strategy was as follows:

#1 Search: (“Lung Neoplasm”[Mesh]) OR ((((((((((((((((((((Lung Neoplasm[Title/Abstract]) OR (Neoplasms, Lung[Title/Abstract])) OR (Neoplasm, Lung[Title/Abstract])) OR (Neoplasms, Pulmonary[Title/Abstract])) OR (Neoplasm, Pulmonary[Title/Abstract])) OR (Pulmonary Neoplasm[Title/Abstract])) OR (Lung Cancer[Title/Abstract])) OR (Cancer, Lung[Title/Abstract])) OR (Cancers, Lung[Title/Abstract])) OR (Lung Cancers[Title/Abstract])) OR (Pulmonary Cancer[Title/Abstract])) OR (Cancer, Pulmonary[Title/Abstract])) OR (Cancers, Pulmonary[Title/Abstract])) OR (Pulmonary Cancers[Title/Abstract])) OR (Pulmonary Cancers[Title/Abstract])) OR (Cancer of Lung[Title/Abstract])) OR (Small Cell Lung Cancer[Title/Abstract])) OR (Oat Cell Lung Cancer[Title/Abstract])) OR (Small Cell Cancer Of The Lung[Title/Abstract])) OR (Carcinoma, Small Cell Lung[Title/Abstract])) OR (Oat Cell Carcinoma of Lung[Title/Abstract]))
#2 Search: (“xihuang” [Supplementary Concept]) OR ((xi huang[Title/Abstract]) OR (xihuangwan[Title/Abstract]))
#3 Search: (((((((((randomized controlled trial[Title/Abstract]) OR RCT[Title/Abstract]) OR random[Title/Abstract]) OR randomly[Title/Abstract]) OR random allocation[Title/Abstract]) OR allocation[Title/Abstract]) OR randomized control trial[Title/Abstract]) OR controlled clinical trial[Title/Abstract]) OR clinical trial[Title/Abstract]) OR clinical study[Title/Abstract]
#1 and #2 and #3

### 2.3 Study selection and data extraction

#### 2.3.1 Selection of studies

**Figure 1:**
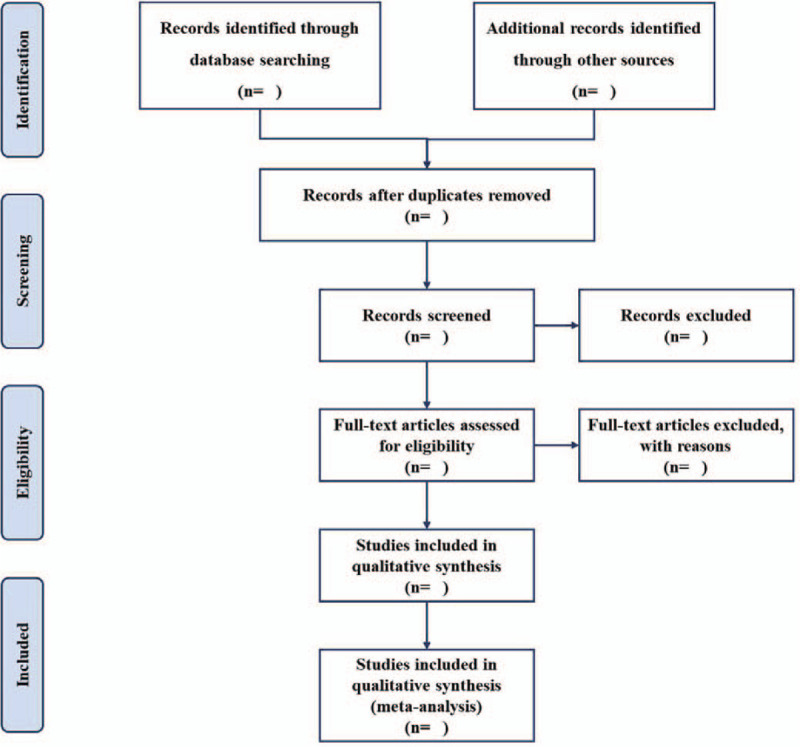
Flow chart of study selection.

The electronic citations extracted out from the above databases will be managed by Endnote X9.0 (Thomson Corporation, CT).^[13]^ First of all, 2 reviewers will independently conduct literature screening, data extraction, and check. In case of disagreements, a third reviewer will participate in consensus conferences. Secondly, we should read the title and abstract of the retrieved research first, exclude the irrelevant literature, and then read the full text of the included research to determine whether it is included. Excluded studies will be listed in a table with reasons. Finally, a PRISMA flow chart (Fig. 1) will be drawn to present the whole process of study selection.

#### 2.3.2 Data extraction and management

For all studies included, 2 authors will independently extract relevant data with the standardized sheet recommended by the Cochrane Handbook of Systematic Reviews of Interventions. The data of those qualified articles will be export to Microsoft Excel, which includes the following items:

(1) Basic information: registered identification, first author, author's unit, country, and publication year.
(2) Research design: sample size, random sequence generation, allocation concealment, analysis of the data, processing of missing data, blinding of the participants, blinding of the outcome measurement, and blinding of the assessors.
(3) Participants: disease, age, disease stage, and diagnostic criteria.
(4) Details of treatment and comparison: delivery methods, dosage, and frequency.
(5) Outcomes: outcome measurement.
(6) Adverse events, conflicts of interest, and other essential information.

#### 2.3.3 Adverse events, conflicts of interest, and other essential information

Regarding missing data of eligible studies, we will contact primary authors to achieve it whenever possible. Any unresolved disagreements between 2 authors will be solved through discussion with another senior author.

#### 2.3.4 Assessment of risk of bias

A tool introduced in the Cochrane Handbook for Systematic Reviews of Interventions will be used to assess a broad category of biases. Risk of bias will be independently assessed by 2 authors using the Cochrane tool of risk of bias. The methodologic quality of all the random control trials will be assessed based on the Cochrane Handbook for Systematic Reviews of Interventions, which comprises 7 items: random sequence generation, allocation concealment, blinding of the participants and personnel, blinding of the outcome assessments, incomplete outcome data, selective reporting, and other sources of bias. The bias risk assessment is divided into 3 criteria: “low risk,” “high risk,” or “unclear risk.” If there are different opinions, we discuss them. If there are still differences exist, we would consult the 3rd appraiser. Otherwise, we need to consult with the Cochrane Professional Group for solution.

#### 2.3.5 Synthesis of data

A meta-analysis will be carried through using RevMan 5.4 (The Cochrane Collaboration, Oxford, England) and Stata 16.0 (Stata Corporation, College Station, TX). For continuous data including the time to union, mean difference (MD) with 95% confident intervals (CIs) will be used to evaluate effect size. Dichotomous data comprising of delayed and nonunion rate and complications will be analyzed using risk ratios (RRs) with 95% CIs. Standard mean difference (SMD) will be used when the treatment outcome was measured by different scales in different studies.

#### 2.3.6 Assessment of heterogeneity

Statistical heterogeneity will be identified by *I*^2^ statistics.^[14]^ Acceptable heterogeneity is considered if *I*^2^ ≤ 50% and a fixed-effect model will be applied. Otherwise, obvious heterogeneity is regarded if *I*^2^ > 50%, and a random-effect model will be utilized.

#### 2.3.7 Subgroup analysis

If the necessary data are available, in the case of high heterogeneity, we will conduct subgroup analysis according to the region of the studies, age, stage of the subjects, types of treatments, and different outcomes. We will evaluate the credibility of the subgroup analysis in term of the guidance.^[15]^ If quantitative synthesis is not appropriate due to substantial heterogeneity, then systematic review will be conducted and the results will be displayed in tables and figures.

#### 2.3.8 Sensitivity analysis

Sensitivity analysis will be conducted to identify the robustness of the result and detect whether there are any exceptional studies bringing about an evident heterogeneity. We will exclude each study included in the analysis one by one. Then we will reanalyze and compile the data and compare the difference between the reobtained effects and the original effects. If there is significant statistical heterogeneity, we will use sensitivity analysis to find the reason by eliminating each study one by one.

#### 2.3.9 Assessment of reporting bias

When there are sufficient studies available (normally >10 studies), we will check the reporting bias using funnel plot and Egger regression test.^[16,17]^*P* < .05 is considered to have publication bias.

#### 2.3.10 Grading the quality of evidence

We will assess the quality of evidence using The Grading of Recommendations Assessment, Development and Evaluation (GRADE), a widely used tool in evaluating the quality of assessment.^[18]^ The assessments will be categorized as high quality, medium quality, low quality, and very low quality.

### 2.4 Patient and public involvement

No patient was involved in this protocol for systematic review and meta-analysis.

### 2.5 Ethics and dissemination

This systematic review will not require ethical approval because this is a secondary analysis of published data and there are no data used in our study that are linked to individual patient data. This systematic review will be disseminated through a peer-reviewed publication.

## 3 Discussion

Lung cancer is the most common malignancy both in women and in men. Xihuang pill is a famous Chinese patent medicine for the treatment of lung cancer in clinical practice, and a series of clinical studies have been conducted on it. However, no systematic review related to Xihuang pill for lung cancer has been published currently. In this study, we will conduct systematic review and meta-analysis to provide more evidence on the effectiveness and safety for it. These findings may provide helpful guidance for clinicians in the treatment of lung cancer.

## 4 Amendments

If amendments are needed, we will update our protocol to include any changes in the whole process of research.

## Author contributions

**Conceptualization:** Junwei Wang, Lu Xiong.

**Data curation:** Junwei Wang, Daorui Hou, Jian Xiong, Yahui Peng.

**Formal analysis:** Yahui Peng, Jian Xiong.

**Funding acquisition:** Lu Xiong.

**Investigation:** Jian Xiong, Yahui Peng, Daorui Hou.

**Methodology:** Junwei Wang, Daorui Hou, Lu Xiong.

**Project administration:** Lu Xiong.

**Resources:** Junwei Wang, Jian Xiong, Yahui Peng.

**Software:** Junwei Wang, Daorui Hou, Jian Xiong.

**Supervision:** Lu Xiong.

**Writing – original draft:** Junwei Wang, Daorui Hou, Jian Xiong.

**Writing – review & editing:** Junwei Wang, Daorui Hou, Jian Xiong, Yahui Peng, Lu Xiong.

## Abbreviations

Abbreviations: CIs = confidence intervals, GRADE = Grading of Recommendations Assessment, Development and Evaluate system, RCTs = randomized controlled trials, SMD = standard mean difference.

## References

[1] Bray F Ferlay J Soerjomataram I. Global cancer statistics 2018: GLOBOCAN estimates of incidence and mortality worldwide for 36 cancers in 185 countries. CA Cancer J Clin 2018;68:394–424. 30207593 10.3322/caac.21492

[2] Evans M. Lung cancer: needs assessment, treatment and therapies. Br J Nurs 2013;22:S15–6. s18, s20–12. 10.12968/bjon.2013.22.Sup17.S15 24067269

[3] Lemjabbar-Alaoui H Hassan OU Yang YW. Lung cancer: biology and treatment options. Biochim Biophys Acta 2015;1856:189–210. 26297204 10.1016/j.bbcan.2015.08.002 PMC4663145

[4] Liao YH Li CI Lin CC. Traditional Chinese medicine as adjunctive therapy improves the long-term survival of lung cancer patients. J Cancer Res Clin Oncol 2017;143:2425–35. 28803328 10.1007/s00432-017-2491-6 PMC11819392

[5] Guo Q Lin J Liu R. Review on the Applications and Molecular Mechanisms of Xihuang Pill in Tumor Treatment. Evid Based Complement Alternat Med 2015;2015:1–6. 10.1155/2015/854307 PMC4479127 26170886

[6] Li C Chen W Zhang M. Modulatory effects of Xihuang Pill on lung cancer treatment by an integrative approach. Biomed Pharmacother 2020;130:1–9. 10.1016/j.biopha.2020.110533 32739739

[7] Su L Jiang Y Xu Y. Xihuang pill promotes apoptosis of Treg cells in the tumor microenvironment in 4T1 mouse breast cancer by upregulating MEKK1/SEK1/JNK1/AP-1 pathway. Biomed Pharmacother 2018;102:1111–9. 29710529 10.1016/j.biopha.2018.03.063

[8] Hong R Wu YQ Wu Y. Effects of xihuangwan in assistant treatment of patients with advanced breast cancer. Zhongguo Zhong Yao Za Zhi 2014;39:1120–3. 24956863

[9] Yu D An GY. Clinical effects of Xihuang Pill combined with chemotherapy in patients with advanced colorectal cancer. Evid Based Complement Alternat Med 2017;2017:1–6. 10.1155/2017/5936086 PMC5387817 28458715

[10] Wang L Li H Zu Q. Clinical research of Xihuang Pill on 60 NHL cases treatment combined with CHOP regimen chemotherapy. J Shandong Univ Trad Chin Med 2012;36:313–5.

[11] Liu B Yu S Xing L. Analysis of therapeutical effects of Xihuang Pills with intraarterial intervention chemotherapy on 80 cases of advanced primary hepatic carcinoma. China J Trad Chin Med Pharm 2010;25:947–8.

[12] Moher D Shamseer L Clarke M. Preferred reporting items for systematic review and meta-analysis protocols (PRISMA-P) 2015 statement. System Rev 2015;4:1–9. 25554246 10.1186/2046-4053-4-1 PMC4320440

[13] Bramer W Bain P. Updating search strategies for systematic reviews using EndNote. J Med Libr Assoc 2017;105:285–9. 28670219 10.5195/jmla.2017.183 PMC5490709

[14] Higgins JP Thompson SG. Quantifying heterogeneity in a meta-analysis. Stat Med 2002;21:1539–58. 12111919 10.1002/sim.1186

[15] Sun X Briel M Walter SD. Is a subgroup effect believable? Updating criteria to evaluate the credibility of subgroup analyses. BMJ 2010;340:850–4. 10.1136/bmj.c117 20354011

[16] Sutton AJ Duval SJ Tweedie RL. Empirical assessment of effect of publication bias on meta-analyses. BMJ 2000;320:1574–7. 10845965 10.1136/bmj.320.7249.1574 PMC27401

[17] Egger M Davey Smith G Schneider M. Bias in meta-analysis detected by a simple, graphical test. BMJ 1997;315:629–34. 9310563 10.1136/bmj.315.7109.629 PMC2127453

[18] Atkins D Best D Briss PA. Grading quality of evidence and strength of recommendations. BMJ 2004;328:1490–4. 15205295 10.1136/bmj.328.7454.1490 PMC428525

